# Activation of apoplastic sugar at the transition stage may be essential for axillary bud outgrowth in the grasses

**DOI:** 10.3389/fpls.2022.1023581

**Published:** 2022-10-26

**Authors:** Tesfamichael H. Kebrom, Andrew N. Doust

**Affiliations:** ^1^ Cooperative Agricultural Research Center, College of Agriculture and Human Sciences, Prairie View A&M University, Prairie View, TX, United States; ^2^ Center for Computational Systems Biology, College of Engineering, Prairie View A&M University, Prairie View, TX, United States; ^3^ Department of Plant Biology, Ecology and Evolution, Oklahoma State University, Stillwater, OK, United States

**Keywords:** shoot branching, axillary bud, dormancy, apoplastic, symplastic, sugar, tillering

## Abstract

Shoot branches develop from buds in leaf axils. Once formed from axillary meristems, the buds enter a transition stage before growing into branches. The buds may transition into dormancy if internal and environmental factors limit sucrose supply to the buds. A fundamental question is why sucrose can be limiting at the transition stage for bud outgrowth, whereas new buds continue to be formed. Sucrose is transported to sink tissues through symplastic or apoplastic pathways and a shift from symplastic to apoplastic pathway is common during seed and fruit development. In addition, symplastic connected tissues are stronger sinks than symplastically isolated tissues that rely on sugars effluxed to the apoplast. Recent studies in sorghum, sugarcane, and maize indicate activation of apoplastic sugar in buds that transition to outgrowth but not to dormancy, although the mode of sugar transport during bud formation is still unclear. Since the apoplastic pathway in sorghum buds was specifically activated during bud outgrowth, we posit that sugar for axillary bud formation is most likely supplied through the symplastic pathway. This suggests a key developmental change at the transition stage, which alters the sugar transport pathway of newly-formed buds from symplastic to apoplastic, making the buds a less strong sink for sugars. We suggest therefore that bud outgrowth that relies on overflow of excess sucrose to the apoplast will be more sensitive to internal and environmental factors that enhance the growth of sink tissues and sucrose demand in the parent shoot; whereas bud formation that relies on symplastic sucrose will be less affected by these factors.

## Introduction

**Figure 1:**
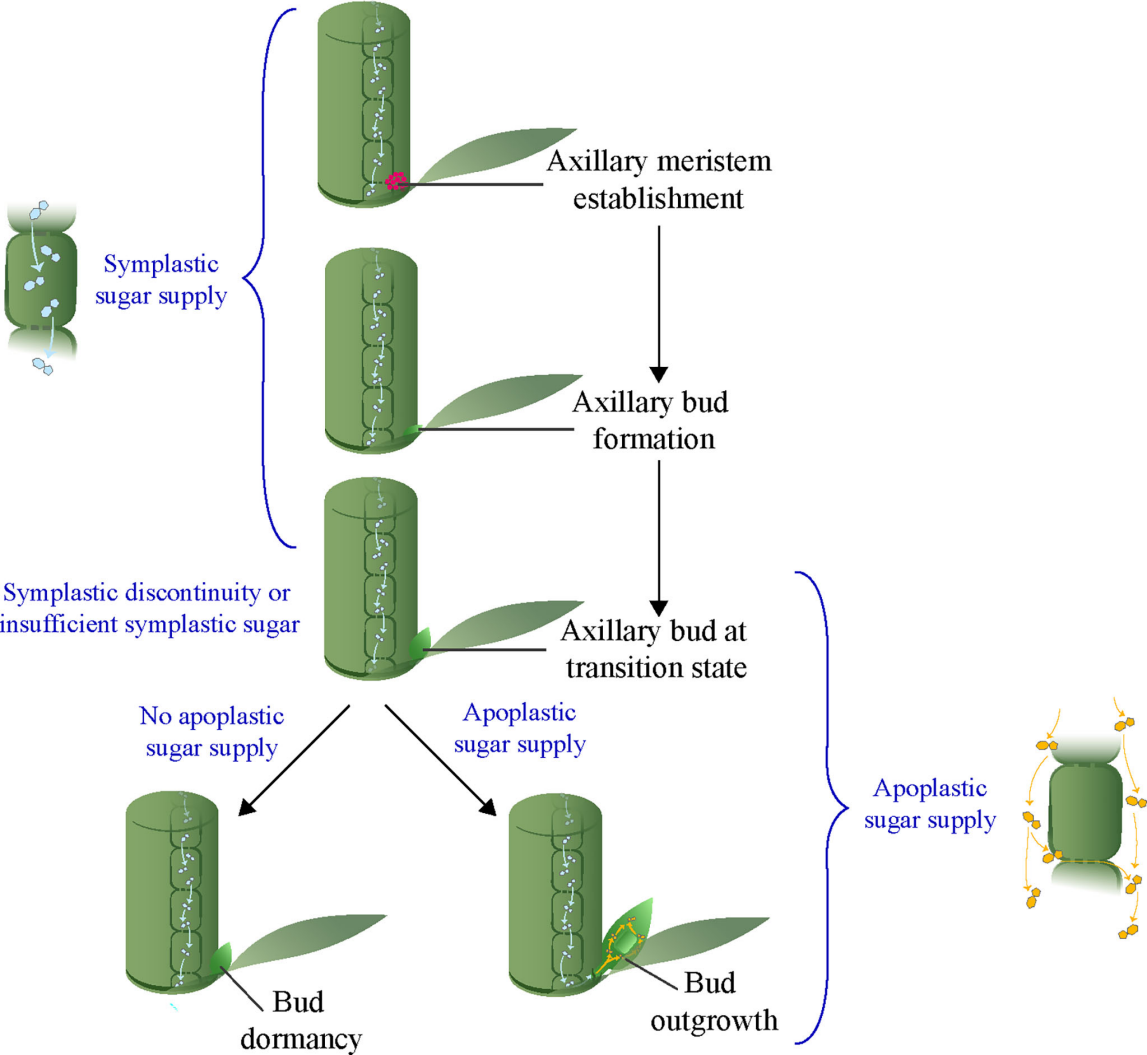
A model of axillary meristem fate depending on availability of apoplastic sugar at the transition stage.

The shoot architecture of annual plants depends on the growth and arrangement of primary and higher order axillary shoots that develop from buds in the leaf axils (Sussex & Kerk, 2001). The first stage in axillary bud initiation is the formation of a few meristematic cells in a stem or subapical tissue of a parent shoot, just above the point of insertion of a leaf or leaf primordia (Grbic & Bleecker, 2000). This leads to a dome of tissues, generated by the meristematic cells, that protrudes from the stem of the parent shoot in a leaf axil, and is the earliest sign of a developing axillary bud in annual plants (**Figure 1**). Subsequent growth of this dome of tissues leads to the formation of a fully developed axillary bud consisting of an embryonic shoot enclosed by young small leaves, and attached at its base to the parent shoot. In most annual plant species, buds are normally formed in each leaf axil regardless of endogenous and environmental conditions. Once the bud is formed, the second step is the outgrowth of the bud into a branch. However, if endogenous and environmental factors are not favorable, a bud may abruptly stop growing and transition into dormancy (Domagalska & Leyser, 2011; Barbier et al., 2019). A fundamental question is why bud outgrowth can be so readily suspended, so that the bud becomes dormant, whereas the formation of a bud is relatively insensitive to internal or environmental conditions.

Previous studies identified a transition stage between the formation of axillary buds and the subsequent development of axillary buds into dormancy or outgrowth (**Figure 1**) (Stafstrom & Sussex, 1992; Shimizu-Sato & Mori, 2001). Since most endogenous and environmental factors that control shoot branching have little influence on axillary bud formation, it is likely that they act at the transition stage to determine the dormancy versus outgrowth fate of axillary buds. Recent studies in different species indicate that sugar supply from the parent shoot to the buds is critical for bud outgrowth (Kebrom et al., 2012; Mason et al., 2014; Barbier et al., 2015; Kebrom & Mullet, 2015; Tarancon et al., 2017; Wang et al., 2021). Sink plant tissues such as developing seeds and fruits are supplied with symplastic and/or apoplastic sugars (Braun et al., 2014). Results that indicate activation of apoplastic sugar during bud outgrowth have been documented in several species in the grasses (Boussiengui-Boussiengui et al., 2016; Kebrom & Mullet, 2016; Dong et al., 2019). However, the mode of sugar supply during bud formation is still unclear, although in the analogous situation of the growth and development of sink plant tissues or organs a transition from symplastic to apoplastic sugar is common (Paniagua et al., 2021). In this review, we posit that the bud transition stage may involve a shift in the axillary bud from relying on symplastic sugar during bud formation to apoplastic sugar for bud outgrowth (**Figure 1**). Such a hypothesis may explain both bud dormancy and bud outgrowth and their reliance on external factors.

In this review, first, we provide a brief summary of how endogenous and environmental factors regulate the dormancy and outgrowth of axillary buds by controlling sugar supply. Next, we review evidence for the activation of apoplastic sugar supply during bud outgrowth, and discuss the possibility of symplastic to apoplastic transition in a developing bud in a manner similar to the transition from symplastic to apoplastic sucrose pathway in developing sink tissues such as seeds and fruits. Finally, we provide a research vision and approaches to investigate mechanisms of sucrose transport during bud formation and outgrowth that will help to understand the transition stage that leads to dormancy versus outgrowth fate of newly-formed axillary buds and shoot branching in annual plants.

## Endogenous and environmental factors directly or indirectly regulate sugar supply to promote axillary bud dormancy or outgrowth

Internal and environmental factors, such as plant hormones and light signals, regulate dormancy and outgrowth of axillary buds and shoot branching in annual plants (Rameau et al., 2015; Barbier et al., 2019; Schneider et al., 2019). A direct or indirect role on dormancy or outgrowth of axillary buds is well established for the plant hormones cytokinins (CK), abscisic acid (ABA), auxin, and strigolactones (SL). Both CK and ABA act within a bud to induce axillary bud outgrowth or dormancy, respectively (Dun et al., 2012; Yao & Finlayson, 2015), whereas auxin acts outside a bud to indirectly induce bud dormancy (Prasad et al., 1993; Booker et al., 2003). Although direct application of SL to a bud induces dormancy (Brewer et al., 2009), it is not precisely known if endogenous SL act within or outside the bud (Stirnberg et al., 2007; Luo et al., 2019). Light signals, such as light enriched with far-red (FR) light or low red (R) light relative to FR, the typical microenvironment of plants growing at high density with mutual shading, also induces axillary bud dormancy and inhibits shoot branching (Kebrom et al., 2006; Finlayson et al., 2010; Drummond et al., 2015). Internal and environmental signals are integrated in part through changes in the expression level of the *Teosinte branched1* (*Tb1*) gene in axillary buds (Wang et al., 2019), with the expression of *Tb1* negatively correlated with sugar levels in the buds (Kebrom et al., 2012; Mason et al., 2014).

Analysis of the sugar levels in axillary buds through direct measurement of sugars or the expression of sugar responsive genes indicates a link between dormancy of newly-formed axillary buds and reduced sugar levels in the buds. For example, a direct measurement of sucrose in wheat revealed a significantly lower level in dormant compared to growing axillary buds, associated with upregulation of the wheat ortholog of the Arabidopsis sugar starvation-inducible *DIN6* gene in the dormant buds (Kebrom et al., 2012). The expression of a *DIN6* ortholog was also upregulated in dormant Rose buds (Wang et al., 2021). In sorghum, reducing the photosynthetic leaf area of young plants through defoliation increased the expression of the sorghum *DIN6* ortholog and inhibited bud outgrowth (Kebrom & Mullet, 2015). Defoliation also inhibited bud outgrowth in decapitated pea plants (Mason et al., 2014; Fichtner et al., 2017). In addition, axillary bud dormancy in phytochrome B (phyB) mutant sorghum (unable to respond to R:FR light) was associated with an increase in the expression of the *DIN6* gene in the buds (Kebrom & Mullet, 2016). Tarancon et al. (2017) analyzed public transcriptome data on axillary bud dormancy and outgrowth and identified a link between bud dormancy and lower sugar levels in the buds of both in annual and perennial plants. Therefore, internal and environmental factors that act outside the bud, including auxin, and low R:FR light, enhance the growth of sink tissues and sugar demand in the parent shoot and indirectly limit sugar supply to the buds and induce bud dormancy. By contrast, decapitation and reduced stem growth at low plant density (high R:FR) increase overflow of sugars to the buds and promote bud outgrowth (reviewed in Kebrom, 2017). The role of internal bud signals, especially CKs, is discussed below.

## Activation of apoplastic sugar supply at transition stage promotes bud outgrowth

The link between dormancy and low sugar level in newly-formed axillary buds (Kebrom et al., 2012; Mason et al., 2014; Barbier et al., 2015; Kebrom & Mullet, 2016; Tarancon et al., 2017; Wang et al., 2021), suggests that internal and environmental factors that control shoot branching regulate sugar supply at the transition stage, just after the bud is formed and before it either progresses to sustained growth to form a branch or transitions into dormancy. Sugars synthesized in leaves are transported as sucrose to non-photosynthetic sink tissues (Braun et al., 2014). There are two main ways that sucrose can be transported to the bud, either cell to cell through plasmodesmata (symplastic pathway), or through efflux of sucrose to the extracellular space (apoplast) from which it enters into destination (sink) cells through the cytosolic membrane (apoplastic pathway) (Lemoine et al., 2013; Braun et al., 2014). Sugar Will Eventually be Exported Transporters (SWEETs) that belong to the Clade III subfamily mediate sucrose efflux from phloem to the apoplast (Eom et al., 2015; Ji et al., 2022). Apoplastic sucrose is transported into the cells by sucrose transporters (SUTs, also known as SUC) or cleaved by cell wall invertases (CWINs) into hexoses to be imported by transmembrane hexose transporters across the cell membrane into cells (Eom et al., 2015; Julius et al., 2017). CWINs are involved in apoplastic sugar transport into sink organs such as developing stem internodes, seeds, and fruits (Bihmidine et al., 2013; Jameson et al., 2016; Ruan, 2022). *CWIN* and *SWEET* gene expression or activities during bud outgrowth have also been identified recently in sugarcane, maize, and sorghum.

Sugarcane is propagated vegetatively through axillary buds in stem cuttings that develop into new shoots. A dramatic increase in CWIN enzyme activity is among the earliest changes during bud outgrowth in sugarcane (Boussiengui-Boussiengui et al., 2016). *CWIN* genes were also upregulated in growing buds of the highly branched *tb1* and *gt1* maize mutants relative to dormant buds in the unbranched wild-type (WT), indicating a role for CWIN in growing maize buds (Dong et al., 2019). In addition, a transcriptome study in sorghum revealed a role for CWINs during the transition stage of newly-formed axillary buds. The buds in the first leaf axil of phyB mutant (*phyB-1*) and WT sorghum are normally formed (Kebrom et al., 2006), but transcriptome results indicated that the expression of *CWIN* genes was activated in the WT bud that transitions to outgrowth, but not in the *phyB* mutant bud that transitions to dormancy (Kebrom & Mullet, 2016). Interestingly, a sorghum *SWEET* gene was coexpressed with the *CWIN* genes in the buds of WT sorghum (Kebrom & Mullet, 2016). The sorghum *SWEET* gene (sobic.007g191200) is orthologous to the Arabidopsis *AtSWEET15* that mediates efflux of sucrose from the maternal seed coat to the apoplast to be transferred to the embryo (Chen et al., 2015). The coexpression of the sorghum *SWEET* and *CWIN* genes provides additional evidence for the activation of the apoplastic pathway during bud outgrowth. Taken together, the studies in sugarcane, maize and sorghum suggest that sugars for bud outgrowth in these species are delivered through the apoplastic pathway. Interestingly, bud burst in rose is associated with the expression of a gene encoding vacuolar acid invertase (*RhVI*) and sucrose breakdown in the buds under light (Girault et al., 2010). However, in the absence of light the activated bud may not elongate unless it imports additional sugars from nearby stem (Girault et al., 2010; Henry et al., 2011). The correlation between the expression of the apoplastic sucrose transporter *RhSUC2* in the bud and nearby stem and an increase in sugar level in the activated bud suggests apoplastic sugar import for sustained bud growth in rose (Henry et al., 2011). The expression or activities of CWINs in sugarcane, maize, and sorghum and SUC in rose indicates variation between species in the control of apoplastic sugar transport to the buds.

## Cyokinins induce the expression of *CWIN* genes

CK can promote axillary bud outgrowth when applied directly to the bud (Pillay & Railton, 1983; Turnbull et al., 1997). The role of CK in bud outgrowth could be through promoting cell division (Riou-Khamlichi et al., 1999). However, CK also induces the expression of *CWIN* genes, and thus plays a role in the transport of apoplastic sugars into sink tissues (Roitsch & Gonzalez, 2004). The expression of CK and sugar metabolism and responsive genes was consistent with the divergent fates of the buds in the *phyB-1* and WT sorghum at transition stage, with the *phyB-1* mutant showing expression of genes that indicate low CK level and activities and sugar starvation that lead to bud dormancy, while the WT shows high CK level and activities and increased sugar levels (upregulation of *CWINs* and sugar-inducible genes) promoting bud outgrowth (Kebrom & Mullet, 2016). A similar link between CK and *CWINs* was demonstrated during the germination and early seedling growth of pea (Jameson et al., 2016). Thus, the role of CK in shoot branching could be through promoting apoplastic sugar metabolism and transport that is required for bud outgrowth. Consistent with this, direct application of CK to the bud does not always promote bud outgrowth (reviewed in Dun et al., 2006), possibly due to limited sugar in the bud, or in the plant that could be directed to the bud. Roman et al. (2016) suggested that CK promotes bud outgrowth in rose (*Rosa hybrida*) through activating the expression of genes, such as *RhVI*, *RhSUSY*, *RhSUC2*, and *RhSWEET10*, that enhance the sink strength of the bud for sugars. A recent study also showed that CK promotes potato sprout branching through inducing vacuolar invertase activity (Salam et al., 2021). Therefore, it is likely that CK also promotes axillary bud outgrowth in the grasses in part through activating the expression of CWINs and enhancing apoplastic sucrose transport into the buds.

## The transition stage of axillary buds: Is it a transition from symplastic sugar during bud formation to apoplastic sugar during bud outgrowth?

The activation of the apoplastic pathway at the transition stage during bud outgrowth in sorghum, sugarcane, and maize suggests axillary buds are initially formed using sucrose delivered through a non-apoplastic pathway. A transition from symplastic to apoplastic sugar is a common phenomenon during the development of other sink tissues such as seeds and fruits (Paniagua et al., 2021). For example, a symplastic discontinuity at the placenta tissue that function as a conduit for assimilate transport from fruit to seed during seed development coincides with the expression of the tomato *CWIN* gene *LIN5* (Palmer et al., 2015; Ruan, 2022). Also, the onset of fruit ripening in grape berry is associated with a transition from the symplastic to the apoplastic pathway (Zhang et al., 2006). Although no prior research, to our knowledge, has demonstrated the mechanisms of sugar supply during axillary bud formation, it is plausible to hypothesize that buds are formed using sugar delivered through the symplastic system and that this transitions to the apoplastic system during bud outgrowth. Interestingly, plant tissues that receive sugars through the symplastic pathway, such as root tips and young leaves, are stronger sinks than symplastically isolated sink tissues that rely on apoplastic sugar (reviewed in Ayre, 2011). This suggests that the processes of bud formation that rely on symplastic sugars are stronger sinks than those of bud outgrowth, and are therefore less sensitive to endogenous and environmental factors that affect sugar production and partitioning. Based on the available information, we suggest a model where, prior to the transition stage, during axillary meristem initiation and bud formation, sugars are delivered through the symplastic system. However, symplastic discontinuity between bud and adjacent cells of the parent shoot or insufficient sugar supply through the symplastic system from the parent shoot at transition stage could lead to suspension of growth and bud dormancy, unless there is sufficient apoplastic sugar transport to allow for bud outgrowth (**Figure 1**).

## Conclusion and future perspectives

A branch develops from a bud through a two-step process: axillary bud formation and bud outgrowth. Internal and environmental factors such as plant hormones and light that control shoot branching can induce bud outgrowth or dormancy indirectly by increasing or decreasing sugar supply from the parent shoot to the newly-formed buds; whereas bud formation is less sensitive to the overall sugar dynamics, including sugar production and demand, in the parent shoot regulated by these factors. For example, auxin and shade signals increase the growth of stem internodes in the parent shoot and indirectly reduce the flow of sugars to the buds. The question is why an increase in sugar demand in the main shoot inhibits the process of bud outgrowth but not bud formation. Activation of apoplastic sugar supply during bud outgrowth in sugarcane, sorghum, and maize suggest that sugar supply during bud formation has to be through the symplastic pathway. Plant tissues supplied with symplastic sugars are stronger sink than tissues that rely on excess sucrose from the parent shoot effluxed to the apoplast. Therefore, it appears that a key developmental change during the transition stage, from bud formation to bud outgrowth, requires an alteration in the mode of sugar supply from symplastic to apoplastic. This change could be the basis for the sensitivity of bud outgrowth to sugar limitations. However, although sugar can be limiting for bud outgrowth in eudicots such as pea and Arabidopsis, the activation of an apoplastic pathway during the transition stage has not yet reported in these species. In addition, an important question is whether the symplastic transport system in the grasses suffers either a discontinuity or is simply insufficient to supply the necessary volumes of sugar for bud outgrowth. Therefore, it is possible that both symplastic and apoplastic sugar transport co-exist during bud outgrowth. Research on the mode of sugar supply during bud formation and outgrowth in both monocots and eudicots appears to be important to understand the regulation of shoot branching in plants.

Significant progress has been made in understanding sugar transport during the growth and development of reproductive organs. The methods developed, including the use of carboxyfluorescine to investigate symplastic connection between source and sink tissues and symplastic domains within sink tissues, could enormously benefit and facilitate the research on sugar transport during bud formation and outgrowth. Also, electron microscopy could be used to study plasmodesmata connection and density, the status of vascular connection, and formation of apoplastic space during the development of bud tissues. A role for the TCP transcription factor BRC1b in restricting plasmodesmata development in aerial axillary buds of potato identified recently is a major advance in understanding how sugar transport could determine the dormancy versus outgrowth fate of axillary buds (Nicolas et al., 2022). In perennial plants, bud dormancy is induced in response to short days by callose deposition in plasmodesmata, which restricts symplastic cell-cell signaling and metabolite flux in the shoot apical meristem (van der Schoot & Rinne, 2011). In a recent study, Paterlini et al. (2021) investigated if an increase in callose synthesis would inhibit axillary bud outgrowth in Arabidopsis. Using the *iclas3* system, the authors increased callose biosynthesis in Arabidopsis companion cells and phloem parenchyma cells, and investigated the effect on axillary bud growth in intact and decapitated excised inflorescence stem sections containing one or two axillary buds. Their results showed that an increase in callose accumulation did not affect bud activation (bud outgrowth), whereas subsequent growth rate of the activated buds was slightly reduced (Paterlini et al., 2021). This implies both that the apoplastic pathway must have been used to supply sugars to the developing bud, and that this pathway is less efficient than symplactic connections. Such methods could be developed for use in other species. Axillary buds are minute and thus it may not be easy to analyze the metabolic and proteomic status, whereas, transcriptome analysis can be conducted easily. However, although there are have been several transcriptomic studies of dormancy and outgrowth of axillary buds in annuals, there are noticeable differences in expression of sugar transport related genes compared to the studies in sorghum and maize. This may be because the transition stage in eudicots is narrow and thus may not be captured easily. Thus, precise determination of the developmental status of buds sampled for transcriptome analysis using biomarker genes for dormancy or growth, as has been done in sorghum and maize studies (Kebrom & Mullet, 2016; Dong et al., 2019), may resolve these issues.

## Author contributions

TK and AD conceived the idea and wrote the paper. All authors contributed to the article and approved the submitted version.

## Funding

This work was supported by Agriculture and Food Research Initiative (AFRI) grant number 2021-67014-33755 from the USDA National Institute of Food and Agriculture, by the Texas A&M System Chancellor’s Research Initiative for the Center for Computational Systems Biology and the USDA-NIFA Evans- Allen funds at the Prairie View A&M University. Funding for AND provided by NSF grant IOS-1938093 and Oklahoma Center for the Advancement of Science grant PS20-002-2.

## Conflict of interest

The authors declare that the research was conducted in the absence of any commercial or financial relationships that could be construed as a potential conflict of interest.

## Publisher’s note

All claims expressed in this article are solely those of the authors and do not necessarily represent those of their affiliated organizations, or those of the publisher, the editors and the reviewers. Any product that may be evaluated in this article, or claim that may be made by its manufacturer, is not guaranteed or endorsed by the publisher.

## References

[B1] Ayre B. G. (2011). Membrane-transport systems for sucrose in relation to whole-plant carbon partitioning. Mol. Plant 4 (3), 377–394. doi: 10.1093/mp/ssr014 21502663

[B2] Barbier F. F. Dun E. A. Kerr S. C. Chabikwa T. G. Beveridge C. A. (2019). An update on the signals controlling shoot branching. Trends Plant Sci. 24 (3), 220–236. doi: 10.1016/j.tplants.2018.12.001 30797425

[B3] Barbier F. Peron T. Lecerf M. Perez-Garcia M. D. Barriere Q. Rolcik J.. (2015). Sucrose is an early modulator of the key hormonal mechanisms controlling bud outgrowth in Rosa hybrida. J. Exp. Bot. 66 (9), 2569–2582. doi: 10.1093/jxb/erv047 25873679 PMC4986866

[B4] Bihmidine S. Hunter C. T. 3rd Johns C. E. Koch K. E. Braun D. M. (2013). Regulation of assimilate import into sink organs: update on molecular drivers of sink strength. Front. Plant Sci. 4, 177. doi: 10.3389/fpls.2013.00177 23761804 PMC3671192

[B5] Booker J. Chatfield S. Leyser O. (2003). Auxin acts in xylem-associated or medullary cells to mediate apical dominance. Plant Cell 15 (2), 495–507. doi: 10.1105/tpc.007542 12566587 PMC141216

[B6] Boussiengui-Boussiengui G. Groenewald J. H. Botha F. C. (2016). Metabolic changes associated with the sink-source transition during sprouting of the axillary buds on the sugarcane culm. Trop. Plant Biol. 9 (1), 1–11. doi: 10.1007/s12042-015-9158-8

[B7] Braun D. M. Wang L. Ruan Y. L. (2014). Understanding and manipulating sucrose phloem loading, unloading, metabolism, and signalling to enhance crop yield and food security. J. Exp. Bot. 65 (7), 1713–1735. doi: 10.1093/jxb/ert416 24347463

[B8] Brewer P. B. Dun E. A. Ferguson B. J. Rameau C. Beveridge C. A. (2009). Strigolactone acts downstream of auxin to regulate bud outgrowth in pea and arabidopsis. Plant Physiol. 150 (1), 482–493. doi: 10.1104/pp.108.134783 19321710 PMC2675716

[B9] Chen L. Q. Lin I. W. Qu X. Q. Sosso D. McFarlane H. E. Londono A.. (2015). A cascade of sequentially expressed sucrose transporters in the seed coat and endosperm provides nutrition for the arabidopsis embryo. Plant Cell 27 (3), 607–619. doi: 10.1105/tpc.114.134585 25794936 PMC4558658

[B10] Domagalska M. A. Leyser O. (2011). Signal integration in the control of shoot branching. Nat. Rev. Mol. Cell Biol. 12 (4), 211–221. doi: 10.1038/nrm3088 21427763

[B11] Dong Z. Xiao Y. Govindarajulu R. Feil R. Siddoway M. L. Nielsen T.. (2019). The regulatory landscape of a core maize domestication module controlling bud dormancy and growth repression. Nat. Commun. 10, 1–15. doi: 10.1038/s41467-019-11774-w 31444327 PMC6707278

[B12] Drummond R. S. Janssen B. J. Luo Z. Oplaat C. Ledger S. E. Wohlers M. W.. (2015). Environmental control of branching in petunia. Plant Physiol. 168 (2), 735–751. doi: 10.1104/pp.15.00486 25911529 PMC4453797

[B13] Dun E. A. de Saint Germain A. Rameau C. Beveridge C. A. (2012). Antagonistic action of strigolactone and cytokinin in bud outgrowth control. Plant Physiol. 158 (1), 487–498. doi: 10.1104/pp.111.186783 22042819 PMC3252097

[B14] Dun E. A. Ferguson B. J. Beveridge C. A. (2006). Apical dominance and shoot branching. divergent opinions or divergent mechanisms? Plant Physiol. 142 (3), 812–819. doi: 10.1104/pp.106.086868 17093134 PMC1630731

[B15] Eom J. S. Chen L. Q. Sosso D. Julius B. T. Lin I. W. Qu X. Q.. (2015). SWEETs, transporters for intracellular and intercellular sugar translocation. Curr. Opin. Plant Biol. 25, 53–62. doi: 10.1016/j.pbi.2015.04.005 25988582

[B16] Fichtner F. Barbier F. F. Feil R. Watanabe M. Annunziata M. G. Chabikwa T. G.. (2017). Trehalose 6-phosphate is involved in triggering axillary bud outgrowth in garden pea (Pisum sativum l.). Plant J. 92 (4), 611–623. doi: 10.1111/tpj.13705 28869799

[B17] Finlayson S. A. Krishnareddy S. R. Kebrom T. H. Casal J. J. (2010). Phytochrome regulation of branching in arabidopsis. Plant Physiol. 152 (4), 1914–1927. doi: 10.1104/pp.109.148833 20154098 PMC2850038

[B18] Girault T. Abidi F. Sigogne M. Pelleschi-Travier S. Boumaza R. Sakr S.. (2010). Sugars are under light control during bud burst in Rosa sp. Plant Cell Environ. 33 (8), 1339–1350. doi: 10.1111/j.1365-3040.2010.02152.x 20374536

[B19] Grbic V. Bleecker A. B. (2000). Axillary meristem development in arabidopsis thaliana. Plant J. 21 (2), 215–223. doi: 10.1046/j.1365-313x.2000.00670.x 10743661

[B20] Henry C. Rabot A. Laloi M. Mortreau E. Sigogne M. Leduc N.. (2011). Regulation of RhSUC2, a sucrose transporter, is correlated with the light control of bud burst in Rosa sp. Plant Cell Environ. 34 (10), 1776–1789. doi: 10.1111/j.1365-3040.2011.02374.x 21635271

[B21] Jameson P. E. Dhandapani P. Novak O. Song J. (2016). Cytokinins and expression of SWEET, SUT, CWINV and AAP genes increase as pea seeds germinate. Int. J. Mol. Sci. 17 (12):1–13. doi: 10.3390/ijms17122013 PMC5187813 27916945

[B22] Ji J. Yang L. Fang Z. Zhang Y. Zhuang M. Lv H.. (2022). Plant SWEET family of sugar transporters: Structure, evolution and biological functions. Biomolecules 12 (2):1–19. doi: 10.3390/biom12020205 PMC8961523 35204707

[B23] Julius B. T. Leach K. A. Tran T. M. Mertz R. A. Braun D. M. (2017). Sugar transporters in plants: New insights and discoveries. Plant Cell Physiol. 58 (9), 1442–1460. doi: 10.1093/pcp/pcx090 28922744

[B24] Kebrom T. H. (2017). A growing stem inhibits bud outgrowth - the overlooked theory of apical dominance. Front. Plant Sci. 8 1874. doi: 10.3389/fpls.2017.01874 29163599 PMC5671643

[B25] Kebrom T. H. Burson B. L. Finlayson S. A. (2006). Phytochrome b represses teosinte Branched1 expression and induces sorghum axillary bud outgrowth in response to light signals. Plant Physiol. 140 (3), 1109–1117. doi: 10.1104/pp.105.074856 16443694 PMC1400571

[B26] Kebrom T. H. Chandler P. M. Swain S. M. King R. W. Richards R. A. Spielmeyer W. (2012). Inhibition of tiller bud outgrowth in the tin mutant of wheat is associated with precocious internode development. Plant Physiol. 160 (1), 308–318. doi: 10.1104/pp.112.197954 22791303 PMC3440208

[B27] Kebrom T. H. Mullet J. E. (2015). Photosynthetic leaf area modulates tiller bud outgrowth in sorghum. Plant Cell Environ. 38 (8), 1471–1478. doi: 10.1111/pce.12500 25496467

[B28] Kebrom T. H. Mullet J. E. (2016). Transcriptome profiling of tiller buds provides new insights into PhyB regulation of tillering and indeterminate growth in sorghum. Plant Physiol. 170 (4), 2232–2250. doi: 10.1104/pp.16.00014 26893475 PMC4824614

[B29] Lemoine R. La Camera S. Atanassova R. Dedaldechamp F. Allario T. Pourtau N.. (2013). Source-to-sink transport of sugar and regulation by environmental factors. Front. Plant Sci. 4, 272. doi: 10.3389/fpls.2013.00272 23898339 PMC3721551

[B30] Luo L. Takahashi M. Kameoka H. Qin R. Shiga T. Kanno Y.. (2019). Developmental analysis of the early steps in strigolactone-mediated axillary bud dormancy in rice. Plant J. 97 (6), 1006–1021. doi: 10.1111/tpj.14266 30740793 PMC6850044

[B31] Mason M. G. Ross J. J. Babst B. A. Wienclaw B. N. Beveridge C. A. (2014). Sugar demand, not auxin, is the initial regulator of apical dominance. Proc. Natl. Acad. Sci. U.S.A. 111 (16), 6092–6097. doi: 10.1073/pnas.1322045111 24711430 PMC4000805

[B32] Nicolas M. Torres-Perez R. Wahl V. Cruz-Oro E. Rodriguez-Buey M. L. Zamarreno A. M.. (2022). Spatial control of potato tuberization by the TCP transcription factor BRANCHED1b. Nat. Plants 8 (3), 281–294. doi: 10.1038/s41477-022-01112-2 35318445

[B33] Palmer W. M. Ru L. Jin Y. Patrick J. W. Ruan Y. L. (2015). Tomato ovary-to-fruit transition is characterized by a spatial shift of mRNAs for cell wall invertase and its inhibitor with the encoded proteins localized to sieve elements. Mol. Plant 8 (2), 315–328. doi: 10.1016/j.molp.2014.12.019 25680776

[B34] Paniagua C. Sinanaj B. Benitez-Alfonso Y. (2021). Plasmodesmata and their role in the regulation of phloem unloading during fruit development. Curr. Opin. Plant Biol. 64, 102145. doi: 10.1016/j.pbi.2021.102145 34826657 PMC8687135

[B35] Paterlini A. Dorussen D. Fichtner F. van Rongen M. Delacruz R. Vojnovic A.. (2021). Callose accumulation in specific phloem cell types reduces axillary bud growth in arabidopsis thaliana. New Phytol. 231 (2), 516–523. doi: 10.1111/nph.17398 33864687

[B36] Pillay I. Railton I. D. (1983). Complete release of axillary buds from apical dominance in intact, light-grown seedlings of pisum sativum l. following a single application of cytokinin. Plant Physiol. 71 (4), 972–974. doi: 10.1104/pp.71.4.972 16662939 PMC1066154

[B37] Prasad T. K. Li X. Abdelrahman A. M. Hosokawa Z. Cloud N. P. Lamotte C. E.. (1993). Does auxin play a role in the release of apical dominance by shoot inversion in ipomoea-nil. Ann. Bot. 71 (3), 223–229. doi: 10.1006/anbo.1993.1028

[B38] Rameau C. Bertheloot J. Leduc N. Andrieu B. Foucher F. Sakr S. (2015). Multiple pathways regulate shoot branching. Front. Plant Sci 5. doi: 10.3389/fpls.2014.00741 PMC4292231 25628627

[B39] Riou-Khamlichi C. Huntley R. Jacqmard A. Murray J. A. (1999). Cytokinin activation of arabidopsis cell division through a d-type cyclin. Science 283 (5407), 1541–1544. doi: 10.1126/science.283.5407.1541 10066178

[B40] Roitsch T. Gonzalez M. C. (2004). Function and regulation of plant invertases: sweet sensations. Trends Plant Sci. 9 (12), 606–613. doi: 10.1016/j.tplants.2004.10.009 15564128

[B41] Roman H. Girault T. Barbier F. Peron T. Brouard N. Pencik A.. (2016). Cytokinins are initial targets of light in the control of bud outgrowth. Plant Physiol. 172 (1), 489–509. doi: 10.1104/pp.16.00530 27462085 PMC5074613

[B42] Ruan Y. L. (2022). CWIN-sugar transporter nexus is a key component for reproductive success. J. Plant Physiol. 268, 153572. doi: 10.1016/j.jplph.2021.153572 34839101

[B43] Salam B. B. Barbier F. Danieli R. Teper-Bamnolker P. Ziv C. Spichal L.. (2021). Sucrose promotes stem branching through cytokinin. Plant Physiol. 185 (4), 1708–1721. doi: 10.1093/plphys/kiab003 33793932 PMC8133652

[B44] Schneider A. Godin C. Boudon F. Demotes-Mainard S. Sakr S. Bertheloot J. (2019). Light regulation of axillary bud outgrowth along plant axes: An overview of the roles of sugars and hormones. Front. Plant Sci. 10 1296. doi: 10.3389/fpls.2019.01296 PMC6813921 31681386

[B45] Shimizu-Sato S. Mori H. (2001). Control of outgrowth and dormancy in axillary buds. Plant Physiol. 127 (4), 1405–1413. doi: 10.1104/pp.010841 11743082 PMC1540171

[B46] Stafstrom J. P. Sussex I. M. (1992). Expression of a ribosomal protein gene in axillary buds of pea seedlings. Plant Physiol. 100 (3), 1494–1502. doi: 10.1104/pp.100.3.1494 16653149 PMC1075811

[B47] Stirnberg P. Furner I. J. Leyser H. M. O. (2007). MAX2 participates in an SCF complex which acts locally at the node to suppress shoot branching. Plant J. 50 (1), 80–94. doi: 10.1111/j.1365-313X.2007.03032.x 17346265

[B48] Sussex I. M. Kerk N. M. (2001). The evolution of plant architecture. Curr. Opin. Plant Biol. 4 (1), 33–37. doi: 10.1016/S1369-5266(00)00132-1 11163165

[B49] Tarancon C. Gonzalez-Grandio E. Oliveros J. C. Nicolas M. Cubas P. (2017). A conserved carbon starvation response underlies bud dormancy in woody and herbaceous species. Front. Plant Sci. 8, 788. doi: 10.3389/fpls.2017.00788 28588590 PMC5440562

[B50] Turnbull C. G. N. Raymond M. A. A. Dodd I. C. Morris S. E. (1997). Rapid increases in cytokinin concentration in lateral buds of chickpea (Cicer arietinum l) during release of apical dominance. Planta 202 (3), 271–276. doi: 10.1007/s004250050128

[B51] van der Schoot C. Rinne P. L. (2011). Dormancy cycling at the shoot apical meristem: transitioning between self-organization and self-arrest. Plant Sci. 180 (1), 120–131. doi: 10.1016/j.plantsci.2010.08.009 21421354

[B52] Wang M. Le Moigne M. A. Bertheloot J. Crespel L. Perez-Garcia M. D. Oge L.. (2019). BRANCHED1: A key hub of shoot branching. Front. Plant Sci. 10, 76. doi: 10.3389/fpls.2019.00076 30809235 PMC6379311

[B53] Wang M. Perez-Garcia M. D. Daviere J. M. Barbier F. Oge L. Gentilhomme J.. (2021). Outgrowth of the axillary bud in rose is controlled by sugar metabolism and signalling. J. Exp. Bot. 72 (8), 3044–3060. doi: 10.1093/jxb/erab046 33543244

[B54] Yao C. Finlayson S. A. (2015). Abscisic acid is a general negative regulator of arabidopsis axillary bud growth. Plant Physiol. 169 (1), 611–626. doi: 10.1104/pp.15.00682 26149576 PMC4577412

[B55] Zhang X. Y. Wang X. L. Wang X. F. Xia G. H. Pan Q. H. Fan R. C.. (2006). A shift of phloem unloading from symplasmic to apoplasmic pathway is involved in developmental onset of ripening in grape berry. Plant Physiol. 142 (1), 220–232. doi: 10.1104/pp.106.081430 16861573 PMC1557625

